# Learning Curve of Docking Time in Robot-Assisted Radical Prostatectomy with the Hugo RAS System: How Many Procedures to Achieve Efficiency?

**DOI:** 10.3390/jcm15093509

**Published:** 2026-05-04

**Authors:** Andrea Iannuzzi, Alberto Ragusa, Alessandro De Giuseppe, Francesco Prata, Francesco Tedesco, Benito Fabio Mirto, Fabio Machiella, Gianluca Muto, Donato Dente, Giovanni Muto, Rocco Papalia

**Affiliations:** 1 Research Unit of Urology, Department of Medicine and Surgery, Università Campus Bio-Medico di Roma, 00128 Rome, Italy; a.degiuseppe@unicampus.it; 2 Department of Urology, Fondazione Policlinico Universitario Campus Bio-Medico, 00128 Rome, Italy; alberto.ragusa@policlinicocampus.it (A.R.); f.prata@policlinicocampus.it (F.P.); francesco.tedesco@policlinicocampus.it (F.T.); rocco.papalia@policlinicocampus.it (R.P.); 3 Pineta Grande Hospital, Department of Urology, 81030 Castel Volturno, Italy; fabio.mirto@pinetagrande.it (B.F.M.); fabio.machiella@pinetagrande.it (F.M.); donato.dente@pinetagrande.it (D.D.); 4 Department of Urology, Ospedale San Giovanni Bosco, 10154 Turin, Italy; gianluca.muto@hotmail.it; 5 Department of Urology, Gruppo Villa Maria(GVM), Maria Pia Hospital, 10132 Turin, Italy; giov.muto@gmail.com

**Keywords:** radical prostatectomy, Hugo RAS, RARP, docking time

## Abstract

**Objectives**: Recently, the Hugo RAS System has been introduced on the market and features a modular design comprising four separate, independent arm carts. In this study we aim to identify the number of consecutive robotic-assisted radical prostatectomies (RARP) required to achieve optimal docking time with this new robotic platform. **Methods**: Data from 68 patients who underwent RARP with the New Hugo RAS System were analyzed. A three-arm setting was used in every case. The docking was executed by the same urology resident who had successfully completed the training course as a bed assistant provided by Medtronic at the ORSI Academy in Aalst, Belgium. Statistical analysis included univariate linear regression to evaluate the association between the number of consecutive procedures (independent variable) and docking time (dependent variable). Additionally, a cumulative sum (CUSUM) analysis was conducted to assess the learning curve, identifying the point at which docking time stabilized. **Results**: The analysis included 68 patients. The median “skin to skin” operative time was 198 min (IQR 90–375), with a total console time median of 150 min (IQR 60–335) and a docking time median of 5 min (IQR 4–13). Linear regression analysis showed a significant negative correlation between the number of procedures performed and docking time (*p* < 0.0017), indicating that increased experience correlates with reduced docking time. CUSUM analysis revealed that after the sixth procedure, docking time consistently declined, suggesting that the learning curve for achieving optimal docking time was reached around this point. **Conclusions**: These findings suggest that, despite being a new platform with four independent arms, the Hugo RAS System allows for a brief docking time to be achieved with just a few procedures, thus not impacting the overall duration of the surgical procedure.

## 1. Introduction

Radical prostatectomy (RP) represents one of the cornerstone procedures in urologic oncology and remains the gold standard for the treatment of localized prostate cancer in appropriately selected patients. Over the past two decades, advances in minimally invasive surgery have dramatically transformed the surgical management of prostate cancer (PC), improving perioperative outcomes, postoperative recovery, and functional preservation [1].

Since the first description of robot-assisted radical prostatectomy (RARP) by Binder and Kramer in 2001 [2], robotic platforms have seen rapid adoption in high-volume urology centers. Their enhanced wristed instrumentation, three-dimensional (3D) vision and tremor filtration offer theoretical advantages in precise steps such as apical dissection and vesicourethral anastomosis. These advantages have been associated with reduced blood loss, shorter hospital stay and earlier return to continence compared with historical series of open or laparoscopic radical prostatectomy [3,4,5].

Although the da Vinci^®^ platform has dominated the field for almost two decades, the expiration of its patent has stimulated the development of new robotic systems. Among these, the Hugo™ RAS (Medtronic, USA) and the Versius^®^ Surgical System (CMR Surgical, UK) currently represent the fastest-growing platforms in Western countries. Other solutions such as the Senhance^®^ Surgical System (Asensus Surgical, USA) have contributed to technological diversification, though with a more limited adoption to date. In parallel, several Asian-developed platforms—most notably the Hinotori™ Surgical Robot in Japan, and systems such as MicroHand S and Toumai^®^ in China—are rapidly expanding in local markets and are expected to further increase global competition in the coming years [6,7,8].

The Hugo™ RAS System distinguishes itself through its modular design, composed of four independent arm carts, and its open-console architecture, which enhances collaboration and communication within the surgical team. The modular setup allows for flexible trocar placement tailored to each patient’s anatomy and for procedures across multiple specialties. However, these same features introduce logistical and spatial challenges, particularly during the early adoption phase, when optimal positioning of the robotic arms must be learned to avoid collisions and ensure ergonomic efficiency [9].

The docking process—defined as the time required to position, align, and connect the robotic arms to the trocars—is a crucial step in determining overall operating room efficiency. Prolonged docking may extend operative time and affect surgical workflow. Hence, evaluating the learning curve for docking is essential, especially when introducing new robotic systems such as Hugo™ RAS.

Previous studies on other platforms, such as the da Vinci Xi and Si, have shown that docking time significantly decreases with experience, typically stabilizing after few cases [10,11,12].

In this context, we aimed to evaluate the learning curve associated with the docking phase of the Hugo™ RAS System during RARP. Specifically, we sought to determine the minimum number of consecutive procedures required to achieve an efficient and reproducible docking time, providing objective evidence to guide future implementation of this new robotic platform.

## 2. Materials and Methods

### 2.1. Patient Population and Exclusion Criteria

This is a prospective observational study including consecutive patients undergoing RARP with the Hugo™ RAS system. From July 2023 to September 2024, 68 patients underwent RARP using the Hugo™ RAS robotic platform. Baseline and demographic data were collected, and written informed consent was obtained from all patients. All surgeries were performed by the same experienced surgeon at the Campus Bio-Medico University of Rome. Docking was consistently executed by the same urology resident, who had successfully completed the bed assistant training course provided by Medtronic at the ORSI Academy in Aalst, Belgium.

The following exclusion criteria were applied: documented visceral or bone metastases on imaging—such as abdominopelvic CT, bone scintigraphy, or PSMA PET—poor health status or reduced life expectancy and anesthesiological contraindications. Patients with cN1M0 and cT3–4 N0M0 were included in the study as part of a multidisciplinary approach after discussion with oncologists, radiotherapists, and radiologists.

In accordance with EAU guidelines, patients with low oncological risk did not undergo additional imaging, while patients with ISUP grade >2 underwent total-body CT with bone scintigraphy or, alternatively, a PSMA PET/CT. The indication for pelvic lymph node dissection (PLND) was based on the Briganti and MSKCC nomograms [13,14,15].

### 2.2. Endpoint, Outcomes and Statistical Analysis

The primary endpoint was to evaluate the learning curve associated with the docking process of the Hugo™ RAS system and to determine the minimum number of RP required to achieve a consistent and acceptable docking time.

For the purposes of this study, docking time was defined as the interval beginning after trocar placement and extending through the complete setup of the robotic system. This includes the positioning of the individual robotic arm carts at the patient’s bedside, their anchoring to the previously inserted trocars, and the configuration of the robotic arms with the appropriate tilt and angulation for the procedure.

The body mass index (BMI) was calculated as weight in kilograms divided by height in meters, squared (kg/m^2^), and post-operative complications were reported according to the Clavien-Dindo classification [16].

Continuous data were presented as median and interquartile ranges (IQR), while frequencies and proportions were used for categorical variables. A linear regression analysis was applied to assess the relationship between the number of consecutive surgical procedures (independent variable) and docking time (dependent variable). Additionally, a cumulative sum (CUSUM) analysis was conducted to evaluate the learning curve and identify the point at which docking time stabilized. The overall median docking time of the cohort was used as the reference (target) value. The CUSUM curve was constructed as the cumulative sum of the differences between each individual docking time and this reference value. The inflection point was defined as the change in slope of the curve, indicating the transition from the initial learning phase to a more stable performance phase. No a priori sample size calculation was done, as this is a descriptive analysis of a learning curve; the sample of 68 patients represents all consecutive eligible cases during the study period. The significance threshold was set at *p* < 0.05. STATA (StataCorp. 2021. Stata Statistical Software: Release 17. College Station, TX, USA, StataCorp LLC.) was used for statistical analyses.

### 2.3. Surgical Setup: Trocar Placement and Docking

Following the induction of general anesthesia and the placement of a transurethral catheter, patients were positioned in a steep Trendelenburg position, with a tilt of up to 30°. The first 11 mm optical trocar was inserted along the midline just above the umbilicus, maintaining a maximum distance of 16 cm from the prostate, the designated pelvic target. Pneumoperitoneum was established at a pressure of 12 mmHg to ensure adequate peritoneal distension. A LAP-TAP block was administered as previously described [17].

Under direct visualization, two additional 8 mm robotic ports were placed on the same transverse line, approximately 3 ± 2 cm below the optical trocar and 10 ± 2 cm laterally from it, minimizing the risk of instrument collision. Two laparoscopic ports for the bed assistant were then inserted along the same transverse axis, about 3 ± 2 cm above the optical port, with an inter-port distance of approximately 7 ± 2 cm. A safety distance of at least 2 cm from bony prominences was maintained for all trocars.

Prior to docking, the robotic arms were angled and positioned approximately 45–60 cm from the surgical bed. The bed assistant remained stationed at the patient’s head throughout the procedure.

The optical trocar was set at 180° with a tilt angle of −30°, the right robotic arm was set at 235° with a tilt angle of −30° and the left robotic arm was set at 135° with a tilt angle of −30° [18].

### 2.4. Surgical Technique

RARP was performed using a standard anterior approach. After mobilizing the bladder to access the Retzius space, bladder neck preservation was attempted when anatomically feasible [19]. Urethral length was preserved during the apical dissection to support early continence recovery [20]. Vesico-urethral anastomosis was completed using a large needle driver and a single-knot, running suture technique [21]. Finally, an abdominal drain was positioned.

### 2.5. Post-Operative Management and Follow-Up

The abdominal drain was removed once the output was below 50 mL, and the transurethral catheter was removed on postoperative day 14. According to EAU guidelines, follow-up included PSA testing every 3 months and urological evaluations every 3 to 6 months [1].

## 3. Results

A total of 68 patients were included in the analysis. The median age was 67 years (IQR 53–79), and the median BMI was 27 kg/m^2^ (IQR 23–34). The median Charlson Comorbidity Index (CCI) was 2 (IQR 2–3), and the median preoperative PSA level was 9.5 ng/mL (IQR 0.34–54). At biopsy, 6% of patients (*n* = 4) were classified as ISUP grade group 1, 37% (*n* = 25) as grade group 2, 43% (*n* = 29) as grade group 3 and 14% (*n* = 10) as grade group 4. MRI revealed extracapsular extension (ECE) in 8 patients (12%). Pelvic lymph node dissection was performed in 18% of cases (Table 1).

**Figure 1:**
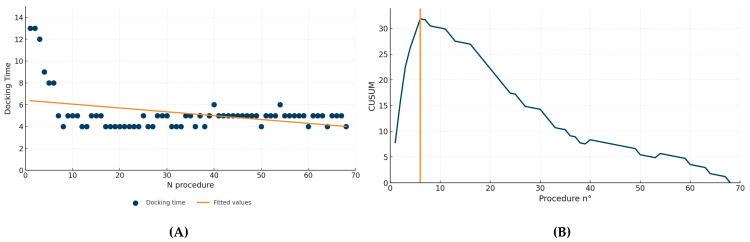
(**A**) Negative linear correlation between the decrease in docking time and the number of consecutive procedures. (**B**) CUSUM curve for docking time. Orange line shows the inflection point of the curve, indicating the end of the learning phase.

The median estimated blood loss was 200 mL (IQR 100–250), and no intraoperative complications were recorded. Postoperative hemoglobin levels had a median of 12 g/dL (IQR 7–15.4). Five patients (8%) experienced Clavien-Dindo grade II complications: two patients required blood transfusion, and three patients developed postoperative fever treated with antibiotics. No major complications occurred. The median length of hospital stay was 3 days (IQR 3–4). The median operative time from incision to closure (“skin to skin”) was 198 min (IQR 90–375), with a median console time of 150 min (IQR 60–335) and a docking time of 5 min (IQR 4–5) (Table 2). Linear regression analysis showed a significant inverse correlation between the number of procedures performed and docking time (*p* < 0.0017, R^2^ = 0.12, β = −0.032 min, 95% CI −0.053 to −0.011), indicating that increased experience was associated with shorter docking times (Figure 1A). CUSUM analysis revealed a consistent reduction in docking time after the sixth procedure, suggesting that the learning curve plateaued at that point (Figure 1B).

## 4. Discussion

The introduction of robotic technology has redefined the surgical treatment of PC, enhancing precision and minimizing morbidity compared to open and laparoscopic approaches. As robotic platforms evolve, new systems such as the Hugo™ RAS System are designed to improve accessibility and versatility in the operating room while maintaining high safety and performance standards. Nevertheless, the transition to modular multi-cart systems introduces new technical and organizational challenges, particularly during the initial docking phase [9].

In our experience with 68 consecutive RARP performed using the Hugo™ RAS System, we observed a median docking time of 5 min (IQR 4–5), representing less than 3% of the total operative time. Importantly, this step proved to be safe, reproducible, and rapidly mastered, with no intraoperative complications or docking-related difficulties. The CUSUM analysis identified a stabilization point after the sixth procedure, indicating that efficient docking could be achieved after a relatively brief learning period. This finding aligns with the concept that the modular nature of Hugo™, while initially demanding greater spatial awareness, allows for rapid adaptation through standardized team coordination.

Our results are consistent with those reported in recent experiences with the Hugo™ RAS platform. Russo et al. recently evaluated 195 consecutive RARPs performed with the Hugo™ RAS platform through a CUSUM analysis, demonstrating that docking time follows a steep and well-defined learning trajectory, with a progressive decline across the early cases until reaching a stable plateau once the operating team becomes familiar with the modular setup of the system [22]. In a series of Hugo procedures with laser-assisted cart positioning, Baunacke et al. described a marked reduction in docking time from 13.5 ± 3.7 to 4.4 ± 0.9 min over the learning curve (*p* < 0.001), underscoring the impact of standardized setup and team training on docking efficiency [23]. In a large monocentric series of 132 RARP performed with Hugo™, Totaro et al. reported a mean docking time of 10 ± 2 min, confirming that docking can be kept within a narrow and reproducible time frame even during the adoption phase [24]. Further Italian and European series have shown comparable brief docking, again highlighting how operative room layout and team experience crucially influence overall efficiency with this modular platform [25,26,27].

Comparative evidence with the da Vinci^®^ system provides additional insight. In the prospective study by Menendez et al., 150 patients were randomized to RARP with either da Vinci^®^ or Hugo™; docking was significantly longer with Hugo™ (18.62 vs. 10.45 min; *p* = 0.02), whereas total operative time and console time were comparable between platforms. Interestingly, Hugo™ outperformed da Vinci^®^ in specific steps such as bladder neck dissection and lymphadenectomy, without differences in intraoperative complications or early postoperative morbidity [28]. Wang et al. in a recent study found that, across eight controlled studies including 1155 patients who underwent RARP, Hugo RAS had a longer docking time than the da Vinci platform (WMD 6.2 min; 95% CI 4.25–8.14; *p* < 0.0001), while no significant differences emerged in total operative time, console time, steps of the procedure, perioperative variables, or oncological and functional outcomes [29].

Taken together, these studies indicate that the modular design of Hugo™—though initially associated with a slightly longer docking—confers several practical advantages. The independent arm carts allow individualized positioning and easier access to the patient, improving ergonomics for both surgeon and assistant. Additionally, the open-console architecture facilitates communication among operating room staff, fostering teamwork and situational awareness, an aspect often overlooked in closed-console systems.

Another relevant aspect concerns training and team dynamics. In our study, docking was performed by a trained urology resident who had completed the official Medtronic course at ORSI Academy. This demonstrates that with adequate preparation, docking can be safely delegated, optimizing workflow and potentially shortening overall operative times. Structured team training programs, similar to those implemented for the da Vinci system, will be essential to ensure reproducibility and safety in multi-cart configurations. Early coordination between the console surgeon and the bedside assistant appears crucial to prevent arm collisions and streamline setup.

From a clinical standpoint, our perioperative results confirm that the introduction of Hugo™ does not compromise patient safety. The low complication rate (8% Clavien-Dindo II), median blood loss of 200 mL, and median hospital stay of 3 days are in line with published series on both da Vinci^®^ and Hugo™ platforms. These findings suggest that modular design, despite initial skepticism, does not increase surgical complexity when properly mastered.

Beyond technical aspects, the Hugo™ RAS System offers potential long-term advantages. Its open-console design supports dual-operator learning and teaching, allowing direct supervision of trainees, and its independent architecture may reduce acquisition and maintenance costs by enabling gradual integration into existing operating rooms. As competition increases, this may help lower institutional costs and expand robotic availability in medium-sized centers. Furthermore, ongoing software updates and compatibility with advanced imaging or AI-guided systems could further enhance precision and workflow in the near future.

Our findings also contribute to the growing body of literature on robotic learning curves in urology. While most research has focused on console performance, our study emphasizes the importance of analyzing ancillary steps—such as docking, trocar placement, and room setup—which, although seemingly minor, play a crucial role in surgical efficiency. Indeed, streamlined docking translates to better operating room turnover and improved cost-effectiveness, which are increasingly relevant in modern health systems.

The use of a single trained docking operator within a consistent surgical team allowed for a controlled assessment of the learning curve by minimizing inter-operator variability. However, the observed improvement over time may also reflect progressive optimization of team coordination and operating room workflow, and should be interpreted within this context.

We acknowledge several limitations of our study. The relatively small sample size and single-center nature may limit generalizability. Finally, our analysis focused exclusively on docking, without assessing other procedural learning curves such as anastomosis or lymphadenectomy. Nevertheless, the consistent trend observed across cases and the objective CUSUM methodology strengthen the validity of our conclusions. Future multicenter prospective trials with larger cohorts and long-term follow-up will be essential to confirm these findings and further define standardized training pathways for the Hugo™ RAS system.

## 5. Conclusions

Despite initial concerns regarding the potential complexity of the docking process due to the use of independent robotic arms, the Hugo™ RAS system proved to be intuitive and manageable. A progressive reduction in docking time was observed, with apparent stabilization after approximately six procedures in this cohort, reflecting a short learning phase under standardized conditions. In this single-center experience, involving a trained surgical team, docking did not significantly prolong overall operative time. These findings support the feasibility of integrating this platform into clinical practice within a structured and trained setting,

## Figures and Tables

**Table 1:** Baseline data.

Variable	Results
Patient (*n*)	68
Age at surgery (years, median, IQR)	67 (53–79)
BMI (kg/m^2^, median, IQR)	27 (23–34)
Charlson Comorbidity Index (median, IQR)	2 (2–3)
PSA (ng/mL, median, IQR)	9.5 (0.34–54)
ISUP grade group at biopsy (*n*, %) - 1 - 2 - 3 - 4 - 5	4 (6) 25 (37) 29 (43) 10 (14) 0
Clinical Stage (*n*, %) - T1c - T2a - T2b - T3_4	31 (45) 24 (35) 5 (8) 8 (12)
ECE at MRI (*n*, %)	8 (12)
Preoperative Hemoglobin (g/dL, median, IQR)	14.2 (9.8–16.5)
Lymphadenectomy (*n*, %)	12 (18)

**Table 2:** Intra- and post-operative outcomes.

Variable	Results
Surgery time skin to skin (minutes, median, IQR)	198 (90–375)
Total console time (minutes, median, IQR)	150 (60–335)
Docking time (minutes, median, IQR)	5 (4–5)
Estimated blood loss (ml, median, IQR)	200 (100–250)
Intraoperative Complications (*n*, %)	0 (0%)
Postoperative Hemoglobin (g/dL, median, IQR)	12 (7–15.4)
Clavien Dindo Complications (*n*, %) - I - II - III - IV	- 0 - 5 (8%) - 0 - 0
Length of Stay (days, median, IQR)	3 (3–4)

## Data Availability

The original contributions presented in the study are included in the article; further inquiries can be directed to the corresponding author.

## Abbreviations

RP	Radical prostatectomy
RARP	Robot-Assisted Radical Prostatectomy
PC	prostate cancer
3D	three-dimensional
PLND	Pelvic Lymph Node Dissection
IQR	interquartile ranges
CUSUM	cumulative sum
BMI	body mass index
ECE	extracapsular extension
CCI	Charlson Comorbidity Index
